# Identification of a novel pyridine derivative with inhibitory activity against ovarian cancer progression *in vivo* and *in vitro*

**DOI:** 10.3389/fphar.2022.1064485

**Published:** 2022-11-18

**Authors:** Lulu Si, Tianjiao Lai, Junru Zhao, Yuxi Jin, Meng Qi, Mingyue Li, Hanlin Fu, Xiaojing Shi, Liying Ma, Ruixia Guo

**Affiliations:** ^1^ Department of Gynecology, The First Affiliated Hospital of Zhengzhou University, Zhengzhou University, Zhengzhou, China; ^2^ Medical Key Laboratory for Prevention and Treatment of Malignant Gynecological Tumor, Zhengzhou, Henan, China; ^3^ State Key Laboratory of Esophageal Cancer Prevention and Treatment, Key Laboratory of Advanced Pharmaceutical Technology, Ministry of Education of China, School of Pharmaceutical Sciences, Zhengzhou University, Zhengzhou, China; ^4^ Laboratory Animal Center, Academy of Medical Science, Zhengzhou University, Zhengzhou, China; ^5^ China Meheco Topfond Pharmaceutical Co., Zhumadian, China; ^6^ Key Laboratory of Cardio-cerebrovascular Drug, Zhumadian, Henan, China

**Keywords:** ovarian cancer, pyridine derivative, histone deacetylase 6, acetylation, cyclin D1

## Abstract

Ovarian cancer is the second leading cause of death of female gynecological malignant tumor patients worldwide. Although surgery and chemotherapy have achieved dramatic achievement, the mortality remains high, resulting in the demand for new specific drug discovery. Disrupting ovarian cancer growth *via* histone deacetylase (HDAC) inhibition is a strategy for cancer therapy or prevention. In this work, we synthesized a novel pyridine derivative named compound H42 and investigated its anti-cancer activity *in vivo* and *in vitro*. We found that compound H42 inhibited ovarian cancer cell proliferation with IC_50_ values of 0.87 μM (SKOV3) and 5.4 μM (A2780). Further studies confirmed that compound H42 induced apoptosis, intracellular ROS production, and DNA damage. Moreover, compound H42 downregulated the expression of histone deacetylase 6 (HDAC6) with a distinct increase in the acetylation of α-tubulin and heat shock protein 90 (HSP90), followed by the degradation of cyclin D1, resulting in cell cycle arrest at the G0/G1 phase. Importantly, ectopic expression of HDAC6 induced deacetylation of HSP90 and α-tubulin, while HDAC6 knockdown upregulated the acetylation of HSP90 and α-tubulin. However, in the nude xenograft mouse study, compound H42 treatment can inhibit ovarian cancer growth without obvious toxicity. These findings indicated that compound H42 inhibited ovarian cancer cell proliferation through inducing cell cycle arrest at the G0/G1 phase *via* regulating HDAC6-mediated acetylation, suggesting compound H42 could serve as a lead compound for further development of ovarian cancer therapeutic agents.

## 1 Introduction

Globally, an estimated 313, 959 new ovarian cancer cases and approximately 207, 252 ovarian cancer deaths occurred in 2020, ranking second in the incidence and death causes among female gynecological malignant tumors (Sung et al., 2021). For all stages of ovarian cancer combined, the overall 5-year survival rate is about 47% (Torre et al., 2018). Due to lack of specific screening and obvious early symptoms, 75% of patients are diagnosed at stage III or IV, for which the 5-year survival rate is about 29% (Reid et al., 2017). Since the 1980s, surgery followed by chemotherapy has become the standard of care in ovarian cancer (Lheureux et al., 2019). Despite 40 years of development of platinum-based first-line chemotherapy, the recurrence rate of women presented with advanced disease reached up to 75% (Lheureux et al., 2019). In recent years, maintenance strategies represented by PARPi have been developed to delay progression and possibly improve overall survival (Lheureux et al., 2015), which are limited by mutations in BRCA. Therefore, given the limitations of these drugs, more specific drugs are required to be developed for ovarian cancer treatment.

Epigenetic gene regulation, mainly including DNA methylation, chromatin remodeling, histone modification, and non-coding RNA regulation, has traditionally been considered closely related to the development and progression of human cancers (Lund and van Lohuizen, 2004; Ramaiah et al., 2021). It should be noted that covalent modification of histones played an important role in posttranslational modifications, which contains methylation, acetylation, ubiquitination, phosphorylation, and others (Zhao and Shilatifard, 2019). Among these, acetylation is often a necessary precursor to other modifications (methylation, phosphorylation, and ubiquitylation) (Yang and Seto, 2008), making it especially important. Acetylation occurring at the histone lysine residues can neutralize the positive charge to disrupt the interaction between histone and nucleosomal DNA, resulting in chromatin opening, thereby promoting active transcription (Zhao and Shilatifard, 2019). It is written by histone acetyltransferases (HATs) and erased by histone deacetylases (HDACs) (Dawson and Kouzarides, 2012; Filippakopoulos and Knapp, 2014). HDACs are involved in posttranslational modification-mediated oncogenic protein fusion and carcinogenic events, which are expressed in various tumors (Glozak and Seto, 2007). HDACs are mainly divided into four classes: HDAC1/2/3/8 (class Ⅰ); HDAC4/5/6/7/9/10 (class Ⅱ); SIRT 1–7 (class Ⅲ); and HDAC11 (class Ⅳ) (Ramaiah et al., 2021). Several kinds of HDACs were also upregulated in ovarian cancer (Yano et al., 2018), making them attractive ovarian cancer therapeutic targets (Takai and Narahara, 2010; Moufarrij et al., 2019).

HDAC6, as the largest member of the HDAC family, has attracted much interest (Pulya et al., 2021) because it can translocate to the cytoplasm and target non-histone substrates, such as α-tubulin (Hubbert et al., 2002), heat shock protein (HSP90) (Kovacs et al., 2005; Liu et al., 2021), and cortactin (Zhang et al., 2007). These diversity substrates make HDAC6 participate in multiple cellular pathways (Hai and Christianson, 2016; Yano et al., 2018; Zhang et al., 2021). It is reported that HDAC6 is required for malignant growth of ovarian cancer cells (Lee et al., 2008) and upregulated in tumors compared to benign lesions (Bazzaro et al., 2008). In particular, high HDAC6 expression was associated with a poor prognosis and chemoresistance in patients with advanced ovarian high-grade serous carcinoma (Yano et al., 2018; Yano et al., 2021).

In this study, we synthesized compound H42 and further evaluated its anti-cancer activity against ovarian cancer. The results showed that compound H42 inhibited ovarian cancer cell proliferation in both A2780 and SKOV3 cells. It induced apoptosis, intracellular ROS production, and DNA damage, along with related-protein expression change. However, compound H42 downregulated the expression of cyclin D1 through regulating HDAC6-mediated acetylation of HSP90, thus leading to G0/G1 phase cell cycle arrest. In addition, compound H42 could repress cancer progression in a human ovarian cancer cell xenograft model without obvious toxicity. These results indicated that compound H42 exerted potent anti-cancer activities *in vivo* and *in vitro*, suggesting it has the potential to be further developed as an agent for ovarian cancer treatment.

## 2 Materials and methods

### 2.1 The synthesis of compound H42

#### 2.1.1 General

For chemical synthesis, all solvents and reagents were purchased from commercial companies. Chemical reactions were monitored by thin-layer chromatography (TLC) and ultra-high performance liquid chromatography-mass spectrometry (UPLC-MS, Water, Milford, MA). In addition, silica gel (200–300 mesh) and silica gel (100–200 mesh) are used for column chromatography. Spectra data of ^1^HNMR and ^13^C NMR were obtained on the DPX-400 NMR (Bruker, Germany), high-resolution mass spectra (HR-MS) data were signed by the Q-Tof type high-resolution mass spectrometer (Bruker Instruments, Inc.), and the melting point of the target compound was determined with the X-5 precision melting point analyzer (Beijing Fukai Instrument Co., Ltd.). The NMR spectra are shown in Supplementary Material.

#### 2.1.2 General process for the synthesis of compound (2)

The 2-aminopyridine (1 mmol) was weighed in a 250-ml round-bottomed flask and dissolved slowly by adding THF (150 ml). N-bromosuccininimide (NBS) (2 × 0.5 mmol) was added in fractions under stirring at room temperature for 0.5 h. The reaction was monitored by TLC (oil ether: ethyl acetate = 5:1). When the reaction was completed, the reaction system was quenched by adding water, extracted three times with ethyl acetate, combined with the organic phase, washed twice with saturated salt water (50 ml × 2), and the organic phase was removed by distillation under reduced pressure to obtain intermediate 2. As the reaction system is relatively simple, intermediate 2 can be directly added into the next step.

#### 2.1.3 General process for the synthesis of compound (3)

Intermediate 2 (1 mmol) was placed in a 100-ml round-bottomed flask, dissolved by adding a mixture of dioxane–water (5:1) solvent. Sequentially, 4-methoxyphenylboronic acid (1.2 mmol) and anhydrous sodium carbonate (2 mmol) were added into the reaction system. Bis(triphenylphosphine) palladium dichloride (0.2 mmol) was added definitively under stirring at room temperature. Thereafter, the system was transferred to an oil bath at 110°C, heated under refluxing for 5 h under nitrogen protection, and monitored by thin-layer chromatography (petroleum ether-ethyl acetate = 5:1). When the reaction of raw material 2 was complete, then we stopped the reaction, and the reaction system cooled down to room temperature, quenched with water, extracted three times with ethyl acetate, combined with the organic phase, washed with saturated salt water, and distilled under reduced pressure to remove the organic phase. Eventually, column chromatography (petroleum ether: ethyl acetate = 3:1) was performed to obtain intermediate 3 as a pale yellow solid.

#### 2.1.4 General process for the synthesis of compound (4)

Then, intermediate 3 (1 mmol) was placed in a 250-ml round-bottomed flask and dissolved by adding tetrahydrofuran solvent. NBS (2 × 0.5 mmol) was added successively, and the reaction was carried out for 0.5 h at room temperature. After the reaction was completed, the organic phase was extracted three times with water and ethyl acetate, combined with the organic phase, washed with saturated salt water, and removed by decompression distillation. Intermediate 4, due to the reaction system being simpler, can be directly added in the next step.

#### 2.1.5 General process for the synthesis of compound (5)

Intermediate 4 was dissolved in a 100-ml round-bottomed flask by adding dioxane–water (5:1) mixture. Then, 4-isopropyl benzene boric acid (1.2 mmol), anhydrous sodium carbonate (2 mmol), and [1,1' -bis (diphenyl phosphine) ferrocene] palladium dichloromethane complex (0.2 mmol) were added. The reaction system was transferred to an oil bath at 110°C, heated and refluxed for 5 h under nitrogen protection, and was stopped by detecting the disappearance of raw material 4 by TLC. The reaction system was cooled to room temperature, quenched with water, extracted three times with ethyl acetate, combined with the organic phase, washed with saturated salt water, and distilled under reduced pressure to remove the organic phase. Last but not the least, column chromatography (petroleum ether: ethyl acetate = 2:1) was used to obtain intermediate 5.

#### 2.1.6 General process for the synthesis of compound (6)

Afterward, intermediate 5 (1 mmol) was placed in 25-ml × 3 round-bottomed flasks and then separately dissolved by adding super dry THF. Sodium hydroxide (2 mmol) was added under stirring at room temperature. After stirring for 0.5 h, methyl 4-bromobenzoyl bromide (1.1 mmol) was added. Finally, the reaction was conducted overnight at room temperature. After the reaction system was completed, the organic phase was extracted three times with water and ethyl acetate, washed with saturated salt water, and removed by decompression distillation. In closing, intermediate six was acquired by column chromatography (petroleum ether: ethyl acetate = 5:1).

#### 2.1.7 General process for the synthesis of compound (7)

Intermediate 6 was placed in a 25-ml round-bottomed flask and dissolved in DCM-MEOH (1:2) solvent. 50% aqueous hydroxylamine (30 mmol) was added under stirring at room temperature for 0.5 h. Then, 10 eq of sodium hydroxide was added. After 10 min, the reaction of raw material 6 was detected by TLC and then, the organic solvent was removed by distillation under reduced pressure. The residue was dissolved by adding water, and dilute hydrochloric acid was added to adjust the pH to 5–6 in an ice bath. The filtered solid was washed with water mixed with petroleum ether-ethyl acetate (1:1) twice and finally dried to obtain white solid end-product 7, *N*-hydroxy-4-{[(5-(4-isopropylphenyl)-3-(4-methoxyphenyl)pyridin-2-yl)amino]methyl}benzamide.

### 2.2 Cell culture and drug preparation

Human ovarian cancer-derived cell lines A2780 and SKOV3 were purchased from Procell Life Science and Technology Co., Ltd. (Wuhan, China) with STR identification. A2780 and SKOV3 cells were cultured in RPMI 1640 (Solarbio, Beijing, China) and McCoy’s 5A (Procell, Wuhan, China), respectively, supplemented with 10% fetal bovine serum (FBS, Gibco, United States) and 1% penicillin–streptomycin at 37°C and 5% CO_2_. Drugs were prepared at a stock concentration of 10 mM with dimethyl sulfoxide (DMSO, sigma, United States). N-acetyl-cysteine was purchased from Dalian Meilun Biotechnlogy Co., LTD, and MG132 was obtained from MedChemExpress.

### 2.3 Cell viability assay

After the cells (5 × 10^3^ per well) were seeded in 96-well plates for 24 h, the culture medium was replaced with fresh medium containing serially 2-fold diluted drugs. Then, the cells were incubated for another 72 h, and the cell viability was measured daily for up to 3 consecutive days using CCK-8 assay. The CCK-8 solution (10 μl) was added to each well, and the absorbance at 450 nm was measured using a microplate reader after the plates incubated at 37°C for 1 h. The half-maximal concentration (IC_50_) was determined using GraphPad Prism 9.3.

### 2.4 Colony formation assay

A2780 cells were seeded in 60-mm dishes at a density of 1, 000 cells per dish. After 24-h incubation, the cells were treated with vehicle or drugs for 14 days until cell colony formation, during which the medium was changed every 3 days. The medium was discarded, and the cells were fixed with 4% paraformaldehyde for 30 min after washing with PBS twice. The cells were stained with 0.1% crystal violet for 30 min and washed with water until the background is clean. After acquiring images, the colonies were counted by ImageJ.

### 2.5 EdU incorporation assay

Cell proliferation was determined by the EdU Cell Proliferation Kit (Beyotime, Shanghai, China) according to the manufacturer’s instruction. Briefly, the cells seeded in 24-well plates were treated with vehicle or drugs for 48 h, EdU solution was added to the medium and incubated for 2 h. The cells were fixed with 4% paraformaldehyde and permeabilized with 0.3% Triton X-100 in PBS. Then, click additive solution was added to the cells and incubated for 30 min at room temperature. The medium was discarded, and the cells were stained with Hoechst 33342. Images were acquired using a fluorescent microscope at 346 nm and 590 nm.

### 2.6 Cell apoptosis assay

Cells were inoculated in 6-well plates, and the culture medium was replaced with fresh medium containing indicated concentration drugs and incubated for 72 h. The cells were collected and apoptotic cells were detected using the Annexin V-FITC Apoptosis Detection Kit (Beyotime, Shanghai, China) by flow cytometry (BD FACSCelesta, United States) according to the manufacturer’s instruction with analysis by FlowJo V10.

### 2.7 Cell cycle assay

Cells were seeded in 60-mm dishes and incubated for 24 h. After the supernatant was replaced with fresh medium without FBS, the cells were incubated for 24 h. The medium was discarded, fresh medium with FBS and indicated concentration drugs were added, and the cells were incubated for another 24 h. Then, the cells were harvested and stained with the staining solution for 30 min at room temperature in the dark according to the manufacturer’s instruction of the cell cycle detection kit (KeyGEN, Nanjing, China). Cell cycle distribution was detected by flow cytometry (BD FACSCelesta, United States) and analyzed by ModfitLT 5.

### 2.8 Wound healing assay

Cells were inoculated into 6-well plates and cultured for 24 h. The cell scratch was made on the plate using a 200-μl pipette tip, and the supernatant was replaced with fresh medium without FBS but containing the indicated concentration of drugs. Then, images were acquired with an inverted microscope to record the scratch area. After 24 h of culture, the scratch area was photographed again. At least five fields were captured in each group. The relative wound healing ratio (%) = (initial scratch area in each group - scratch area after 24 h in each group)/(initial scratch area in each group) × 100%.

### 2.9 Transwell migration assay

Cells in 200 μl medium without FBS were added to the upper chamber, and 600 μl medium with FBS and indicated concentration of drugs were added to the lower chamber, which was cultured for 24 h. After the supernatant was removed, the cells were fixed with 4% paraformaldehyde and stained with 0.1% crystal violet for 30 min. The cells in the upper chamber were wiped with a cotton swab lightly, and the migration cells in the lower chamber were observed and counted via an inverted microscope.

### 2.10 Measurement of intracellular ROS

Intracellular ROS was measured using the Reactive Oxygen Species Assay Kit (Beyotime, Shanghai, China). Briefly, the cells were plated into a 6-well plate and cultured for 24 h. The medium was replaced with fresh medium containing the indicated concentration of drugs and incubated for 48 h. The medium was discarded, and DCFH-DA (10 μM) was added to the cells for 20-min incubation at 37°C. Then, intracellular ROS was measured by flow cytometry (BD FACSCelesta, United States).

### 2.11 Alkaline comet assay

Cells were inoculated into 6-well plates and treated with indicated concentration of drugs for 48 h. The cells were harvested, mixed with low-melting point agarose, and layered onto microscopic slides, which were covered by a glass coverslip and incubated at 4°C for 15 min to allow gels to be solidified. The slides were placed in lysis solution for 2 h at 4°C to allow DNA unwinding. Then, the slides were placed in electrophoresis buffer, which were conducted for 30 min at 300 mA. After rinsing with distilled with water, the slides were dipped in 75% ethanol and dried for 15 min at 37°C. DNA was stained for 10 min at room temperature. The slides were rinsed with distilled water and imaged by a fluorescent microscope.

### 2.12 Indirect immunofluorescence assay

Cells were plated on glass coverslips placed in a 24-well plate. After culturing for 24 h, the cells were treated with indicated concentration of drugs and incubated for another 48 h. Then, the cells were fixed with 4% paraformaldehyde and permeabilized with 0.3% Triton X-100 in PBS. After blocking with 5% BSA in PBS for 30 min, the cells were incubated with phosphor-H2A antibody (1:250, ab81299, Abcam) for 1 h, washed with PBS three times, and incubated with fluorescent secondary antibody (1:2000, Alexa Fluor 488 goat anti-mouse IgG, Abcam) for 1 h. The slides were mounted with DAPI, and the cells were imaged using a fluorescence confocal microscope at 346 nm and 488 nm.

### 2.13 Western blot

Cells were washed with cold PBS twice and lysed with RIPA with protease and phosphorylase inhibitor (Epizyme, Shanghai, China). Proteins were boiled and electrophoretically separated with 10% or 12% polyacrylamide gel at 80–120 V. The proteins were transferred to 0.22-μm or 0.45-μm PVDF membranes (Millipore, Germany), which were blocked with 5% BSA in TBST for 2 h. Then, the membranes were incubated with a primary antibody at 4°C overnight. After washing with TBST three times, the membranes were incubated with HRP-linked secondary antibodies (1:1 000 dilution, CST) and washed with TBST three times and visualized using ECL reagents (Millipore, Germany) by ChemiDOC Imaging System (GE Healthcare Life Sciences, United States).

Antibodies were used against acetyl-α-tubulin (#5335, Cell Signaling, United States), α-tubulin (#2125, Cell Signaling, United States), HDAC6 (#7612, Cell Signaling, United States), caspase-3 (#14220, Cell Signaling, United States), caspase-7 (#12827, Cell Signaling, United States), cleaved caspase-9 (#9508, Cell Signaling, United States), PARP (#9542, Cell Signaling, United States), CDK4 (#12790, Cell Signaling, United States), CDK6 (#3136, Cell Signaling, United States), cyclin D1 (#2978, Cell Signaling, United States), cyclin D3 (#2936, Cell Signaling, United States), pCNA(#13110, Cell Signaling, United States), p-c-Jun (#91952, Cell Signaling, United States), p-p38 (#4511, Cell Signaling, United States), Wee1 (#13084, Cell Signaling, United States), p-CDC25c (#4901, Cell Signaling, United States), γH2AX (ab81299, Abcam, United Kingdom), p-CHK2 (ab32148, Abcam, United Kingdom), p-CHK1 (#2348, Cell Signaling, United States, United States), E-cadherin (#3195, Cell Signaling, United States, United States), N-cadherin (#13116, Cell Signaling, United States), Snail (#5741, Cell Signaling, United States), GAPDH (#5174, Cell Signaling, United States), β-actin (#4970, Cell Signaling, United States), Bcl-2 (#15071T, Cell Signaling, United States), p-Erk1/2 (#18544S, Cell Signaling, United States), H3 (#4499, Cell Signaling, United States), acetyl-H3K9 (#9649, Cell Signaling, United States), HSP90 (60318-1-Ig, Proteintech, United States), and acetyl- HSP90 (ABP50105, Abbkine, United States).

### 2.14 Real-time PCR

After the cells were treated by compound H42 or control for specific time, the total RNA was extracted by TRIzol according to the instructions, followed by cDNA synthesis using the RevertAid First-Strand cDNA Synthesis Kit (K16215, ThermoFisher). The mRNA level of cyclin D1 was measured by real-time PCR with 18s as the control. The sequence of primers was as follows: forward primer of cyclin D1: 5′-CAC​GCG​CAG​ACC​TTC​GT-3′, reverse primer of cyclin D1: 5′-ATG​GAG​GGC​GGA​TTG​GAA-3’; forward primer of 18S: 5′-CGG​CGA​CGA​CCC​ATT​CGA​AC-3′, and reverse primer of 18S: 5′-GAA​TCG​AAC​CCT​GAT​TCC​CCG​TC-3’.

### 2.15 HDAC6 silencing and overexpression

A2780 cells were infected with lentiviruses carrying HDAC6 (shHDAC6) or empty vectors (GeneChem, Shanghai, China). After screening, shHDAC6 A2780 cells were obtained. Over-HDAC6 was produced by transfecting A2780 cells with the HDAC6 overexpression plasmid (GeneChem, Shanghai, China).

### 2.16 Xenograft study

A total of 10 female Balb/c nude mice (4–5 weeks old) were gifted by GemPharmatech Co., Ltd. and housed in the Animal Experimental Center of Henan Province. SKOV3 cells (2 × 10^6^) suspended in 50 μl medium were subcutaneously injected into the right axilla of each nude mouse. When tumor volume was greater than 100 mm^3^, the mice were randomly divided into two groups (*N* = 5 per group): 1) mice that were given H42 at 50 mg/kg once a day; 2) mice that were given PBS once a day. Tumor volume and body weight were measured every 2–3 days. The mouse state was monitored every day. At 19 days after administration, all mice were euthanized and tumor weight was measured.

### 2.17 Statistical analysis

Data were presented as mean ± SD of three independent experiments. Comparisons between two groups were analyzed using the two-tailed unpaired Student’s *t*-test. Multiple group comparisons were analyzed using one-way ANOVA followed by Tukey’s post hoc test. Statistical analyses were performed using GraphPad Prism 9.3. Statistically significant differences were achieved when p-value is less than 0.05. *p ˂ 0.05; **p ˂ 0.01; ***p ˂ 0.001; ****p ˂ 0.0001.

## 3 Results

### 3.1 Chemistry

**FIGURE 1:**
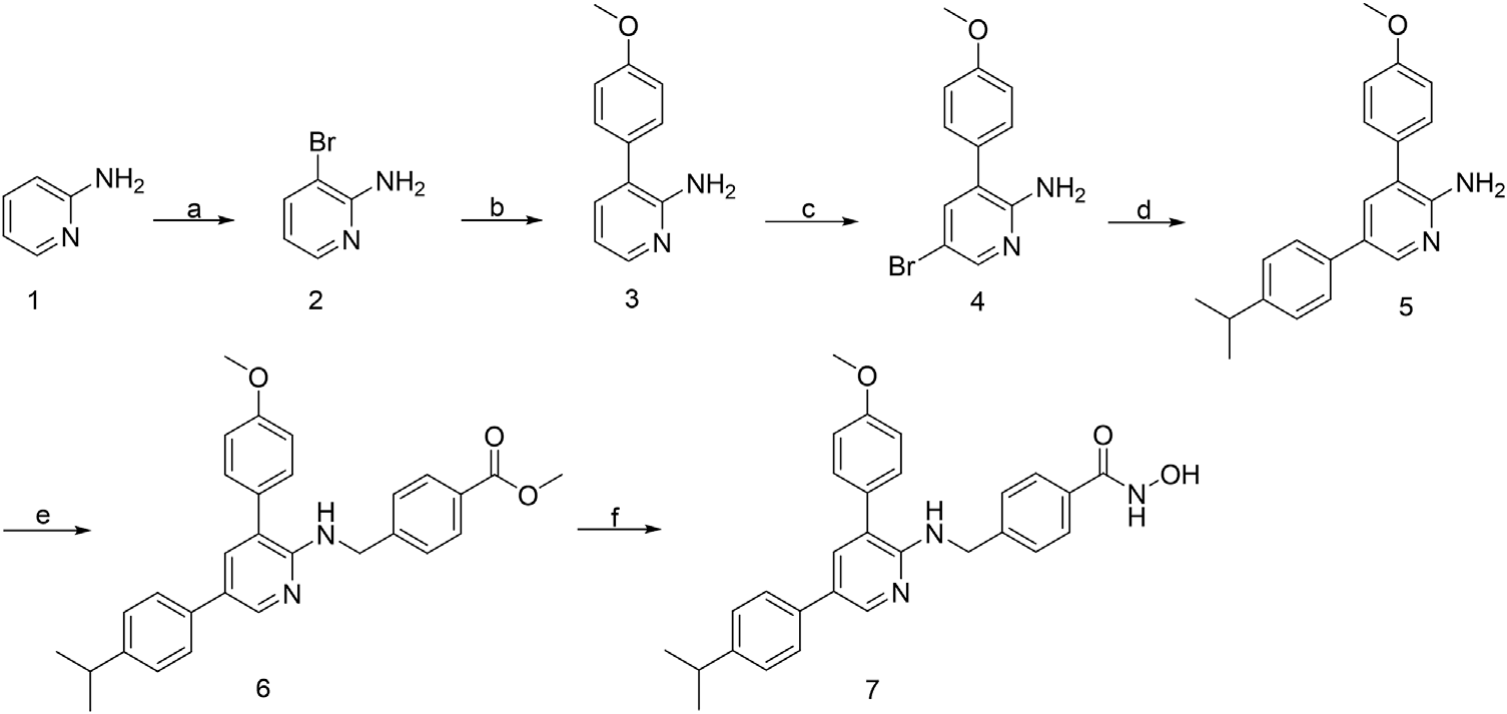
Synthetic route of compound H42. Reagents and conditions: (a) NBS, THF, rt; (b) Na_2_CO_3_, Pd (pph3) 2Cl_2_, 4-methoxyphenylboronic acid, 1,4-dioxane-H_2_O (5:1), 110°C, reflux; (c) NBS, THF, rt; (d) Na_2_CO_3_, Pd (pddf) Cl_2_, 4-isopropylphenylboronic acid, 1,4-dioxane-H_2_O (5:1), 110°C, reflux; (e) NaH, methyl 4-(bromomethyl) benzoate, super dry THF, rt; and (f) 1) hydroxylamine (50% water), NaOH, DCM-MeOH (1:2); 2) formic acid, water. (7) Compound H42: N-hydroxy-4-{[(5-(4-isopropylphenyl)-3-(4-methoxyphenyl)pyridin-2-yl)amino]methyl}benzamide.

From our in-house library screening, we have identified compound H42 as a potent inhibitor against ovarian cancer proliferation. The synthesis of the novel pyridine derivative H42 is illustrated in Figure 1. The compounds 2 and 4 were obtained by substitution reaction with N-bromosuccinimide (NBS) in tetrahydrofuran solution (THF) at room temperature. The intermediates 3 and 5 were obtained through the Suzuki coupling reaction of compounds 2 and 4, respectively, with different substituted phenylboric acid, sodium carbonate, and palladium catalysts in a mixed solvent of dioxane and water. Subsequently, compound 5 was substituted with methyl 4-bromobenzoate in THF to derive intermediate 6. After that, the target compound 7 could be acquired directly from 6 by hydrolysis of hydroxylamine with 75%–82% yield.

### 3.2 Compound H42 inhibited ovarian cancer cell proliferation in a dose- and time-dependent manner

**FIGURE 2:**
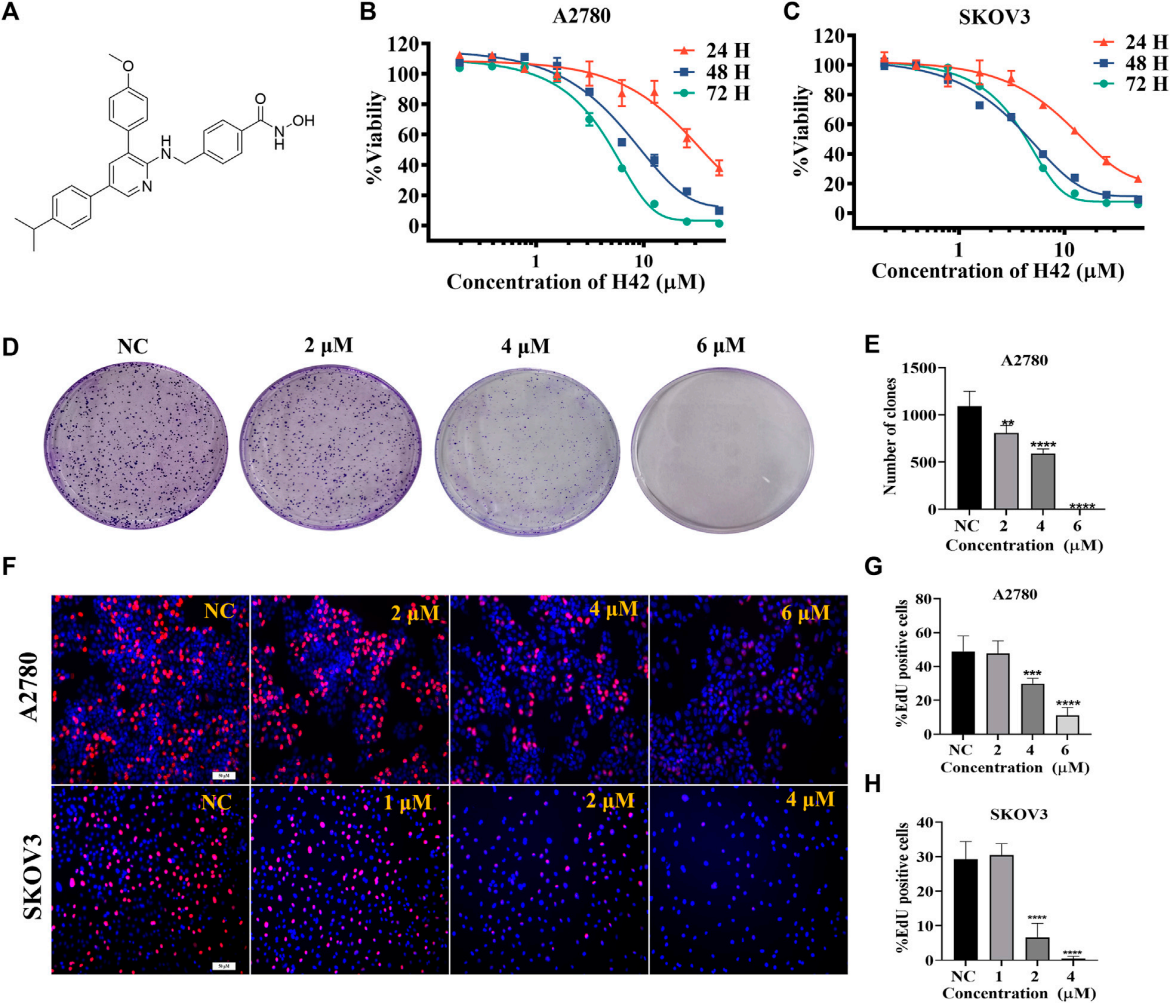
Compound H42 inhibited ovarian cancer cell proliferation *in vitro*. **(A)** Structure of compound H42. The effect of compound H42 in A2780 **(B)** and SKOV3 **(C)** cells in 24, 48, and 72 H was detected by CCK-8. **(D)** Colony formation of A2780 cells treated with different concentrations of compound H42. **(E)** Analysis of colony numbers in various drug concentrations. **(F)**Proliferation of A2780 and SKOV3 cells was probed by EdU staining. Quantification of EdU-incorporated cells in A2780 **(G)** and SKOV3 **(H)** cells. Data are all represented as mean ± SD of at least three independent experiments. **p < 0.01, ***p < 0.001, and ****p < 0.0001 were compared with the control.

To further confirm the inhibitory activity of H42 (Figure 2A) on ovarian cancer, SKOV3 and A2780 cells were treated with H42 at different concentrations, and cell viability was detected by CCK-8 at 24 H, 48 H, and 72 H. The results showed that compound H42 inhibited A2780 cell proliferation with 50% inhibitory concentration (IC_50_) of 28.43 ± 2.13 μΜ (24 H), 8.54 ± 0.93 μΜ (48 H), and 5.40 ± 0.53 μΜ (72 H) inhibited SKOV3 cell proliferation with an IC_50_ of 3.16 ± 0.17 μΜ (24 H), 0.94 ± 0.03 μΜ (48 H), and 0.85 ± 0.02 μΜ (72 H), respectively (Figures 2B,C), suggesting that with the increase of drug concentration and incubation time, the inhibitory effect was enhanced. Then, the effect of compound H42 on growth kinetics of a single ovarian cancer cell was evaluated by colony formation assay. Compound H42 significantly decreased the number of colonies formed in A2780 cells at various concentrations compared to control (Figures 2D,E). To further support the aforementioned observation, the cell proliferation rate was also measured by Edu assay. As shown in Figures 2F–H, compound H42 could reduce the viability of SKOV3 and A2780 cells in a concentration-dependent manner. These results revealed that compound H42 could inhibit ovarian cancer cell proliferation in a dose- and time-dependent manner.

### 3.3 Compound H42 inhibited ovarian cancer cell migration

**FIGURE 3:**
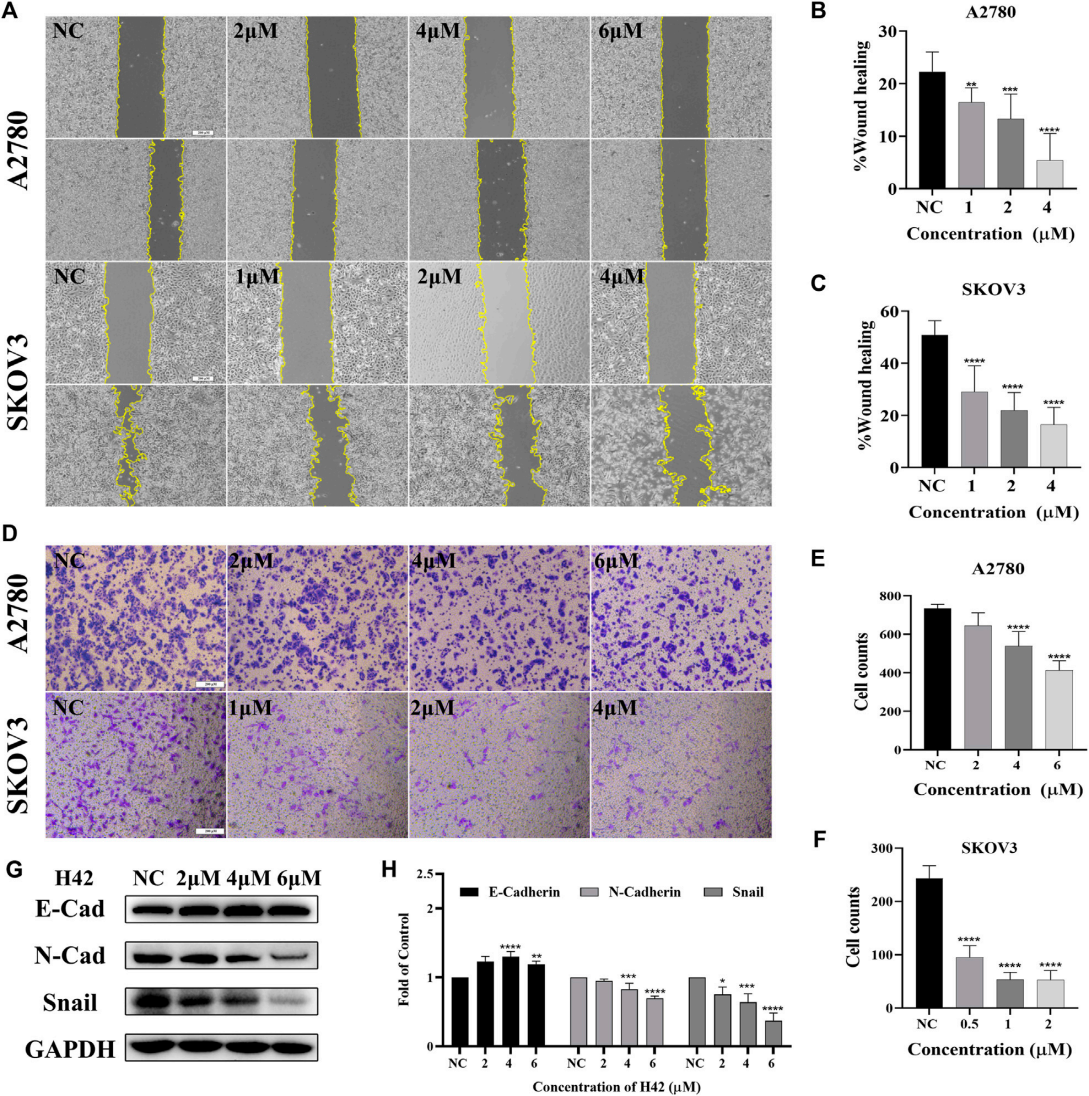
Compound H42 inhibited migration of ovarian cancer cells. **(A–C)** The migration of A2780 and SKOV3 cells treated with various concentrations of H42 was determined by wound healing assay. **(D–F)** Migration of A2780 and SKOV3 cells treated with various concentrations of H42 was determined by transwell assay. (**G**) Expression of migration-related proteins in A2780 cells was measured by Western blot and analyzed in the density ratio (**H**). Data are represented as mean ± SD of at least three independent experiments. *p < 0.05, **p < 0.01, ***p < 0.001, and ****p < 0.0001 were compared with the control.

The effect of H42 on the migration of A2780 and SKOV3 cells was evaluated using wound healing and transwell assay. The results of wound healing assay showed that compared with the control group, H42 treatment inhibited cellular wound healing (Figures 3A–C), and transwell assay results showed that H42 treatment reduced the number of cells migrating to the lower chamber in both A2780 and SKOV3 cells (Figures 3D–F). Moreover, Western blot was used to investigate the expression of metastasis-related proteins. Also, the results showed that the expression of N-cadherin and snail (known to promote metastasis) decreased while the expression of E-cadherin (known to be adverse to metastasis) increased (Figures 3G,H), suggesting that compound H42 inhibited ovarian cancer cell migration.

### 3.4 Compound H42 induced apoptosis of ovarian cancer cells

**FIGURE 4:**
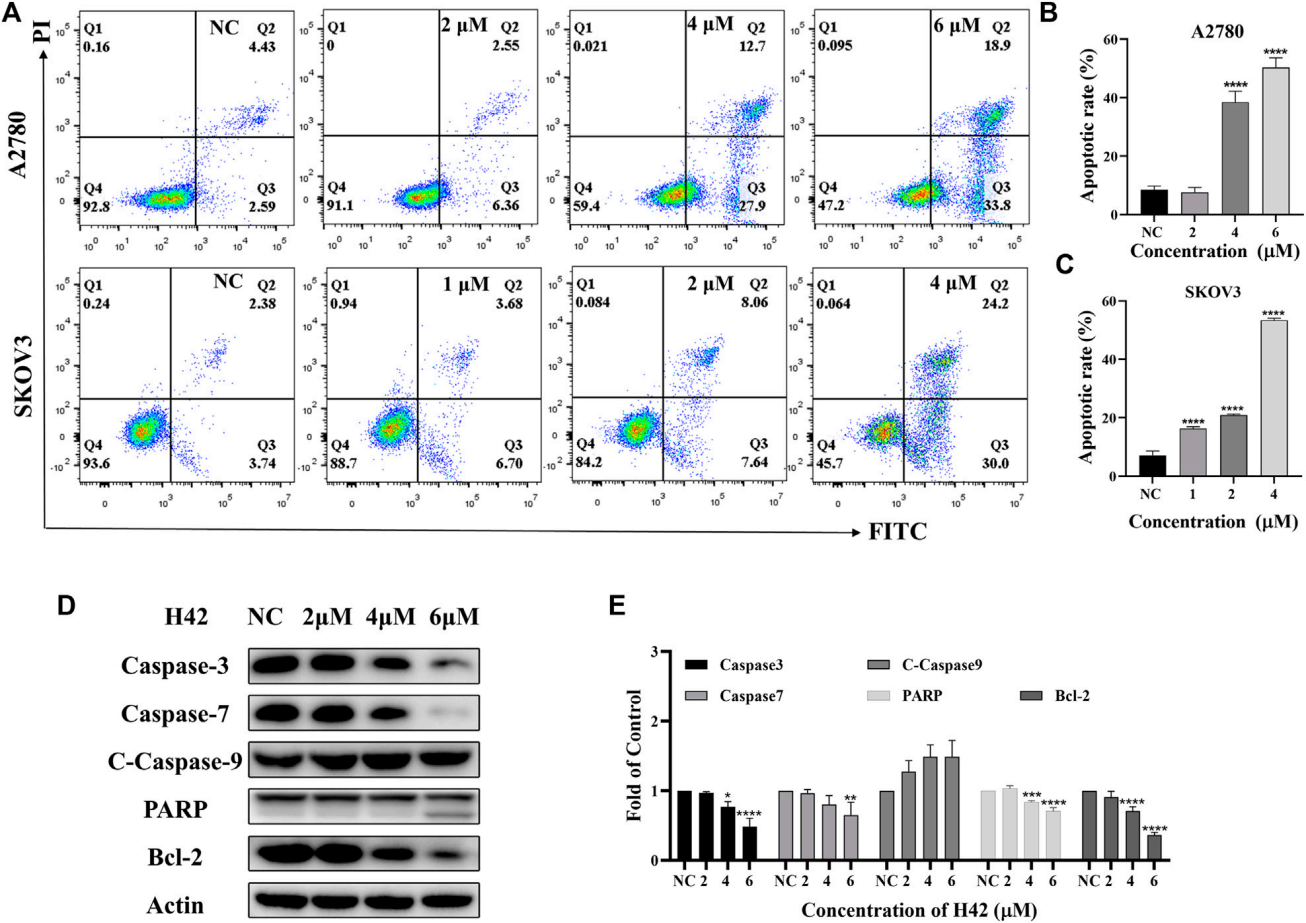
Compound H42 induced apoptosis in A2780 and SKOV3 cells. A2780 and SKOV3 cells were treated with various concentrations of H42 for 72 H. **(A)** Apoptosis of cells were determined by Annexin v-FITC/PI double-staining analysis using FCM and **(B–C)**analyzed by percentage. **(D)** Expression of apoptosis-related proteins in A2780 was measured by Western blot and **(E)** analyzed in the density ratio. Data are all represented as mean ± SD of at least three independent experiments. *p < 0.05, **p < 0.01, ***p < 0.001, and ****p < 0.0001 were compared with the control.

Since inhibition of cell proliferation is often associated with apoptosis, the apoptotic effects of compound H42 on ovarian cancer cells were examined with Annexin V-FITC/PI double staining by flow cytometry. The results showed that with the increasing concentration, compound H42 significantly increased the population of apoptotic cells in both early and late stages compared to the control in both SKOV3 and A2780 cells (Figures 4A–C). We also examined the expression of key proteins involved in apoptosis. The results showed that compound H42 treatment induced a downregulated expression of caspase-3, caspase-7, and PARP, while the expression of cleaved caspase-9 significantly increased after 72 h treatment (Figures 4D,E; Supplementary Figure S2A). Meanwhile, the expression of anti-apoptosis protein Bcl-2 also significantly decreased (Figures 4D,E; Supplementary Figure S2A). These findings indicated that H42 could induce ovarian cancer cell apoptosis potentially *via* the mitochondrion-mediated pathway.

### 3.5 Compound H42 induced oxidative stress in ovarian cancer cells

**FIGURE 5:**
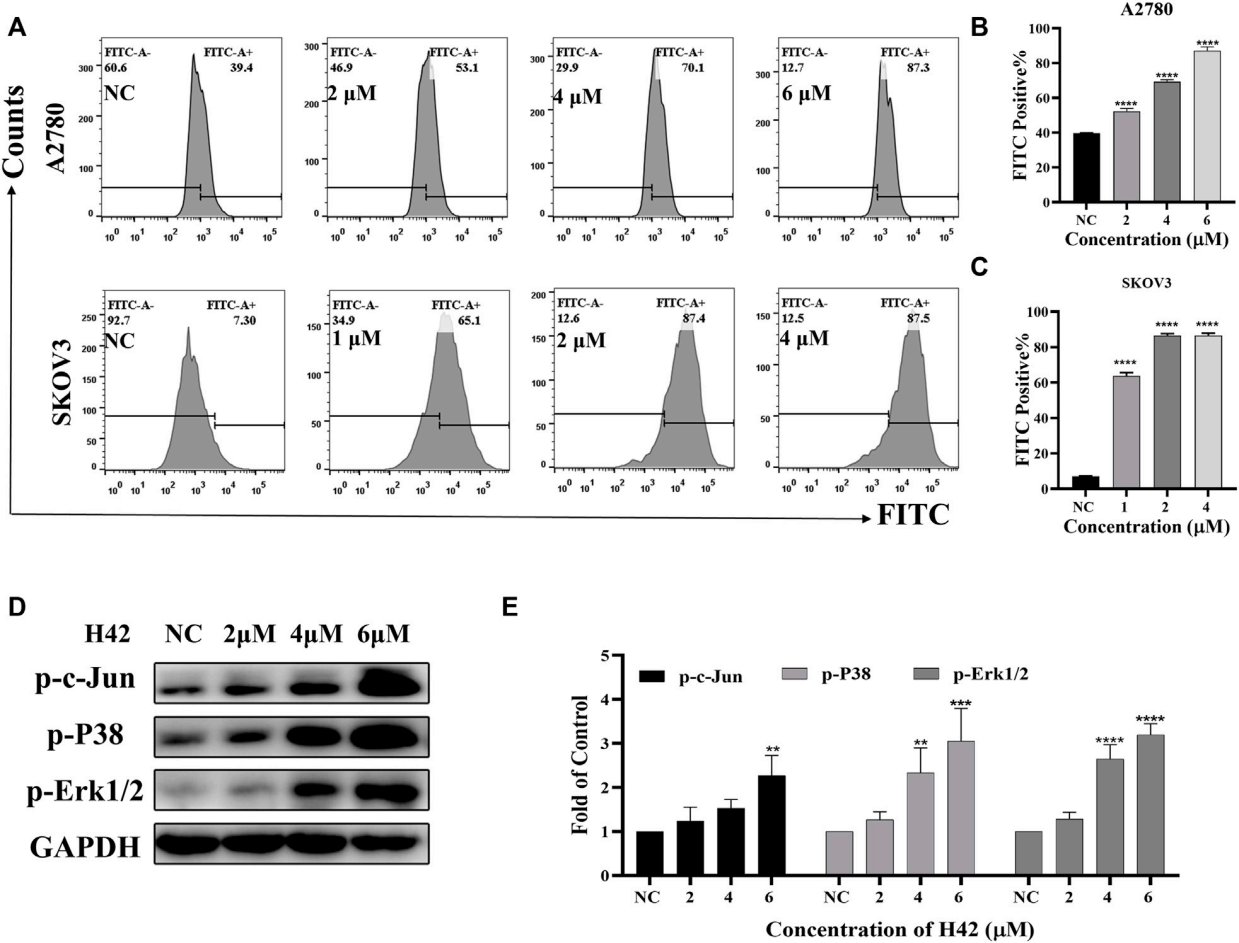
Compound H42 triggered cellular ROS generation in ovarian cancer cells. A2780 and SKOV3 cells were treated with various concentrations of H42 for 48 H. **(A)** Cellular ROS was detected by the DCFH-DA probe using FCM. Quantitative analysis of ROS generation is shown in **(B,C)**. **(D)** Expression of ROS-related proteins in A2780 cells was examined by Western blot and **(E)** analyzed in the density ratio. Data are all represented as mean ± SD of at least three independent experiments. **p < 0.01, ***p < 0.001, and ****p < 0.0001 were compared with the control.

**FIGURE 6:**
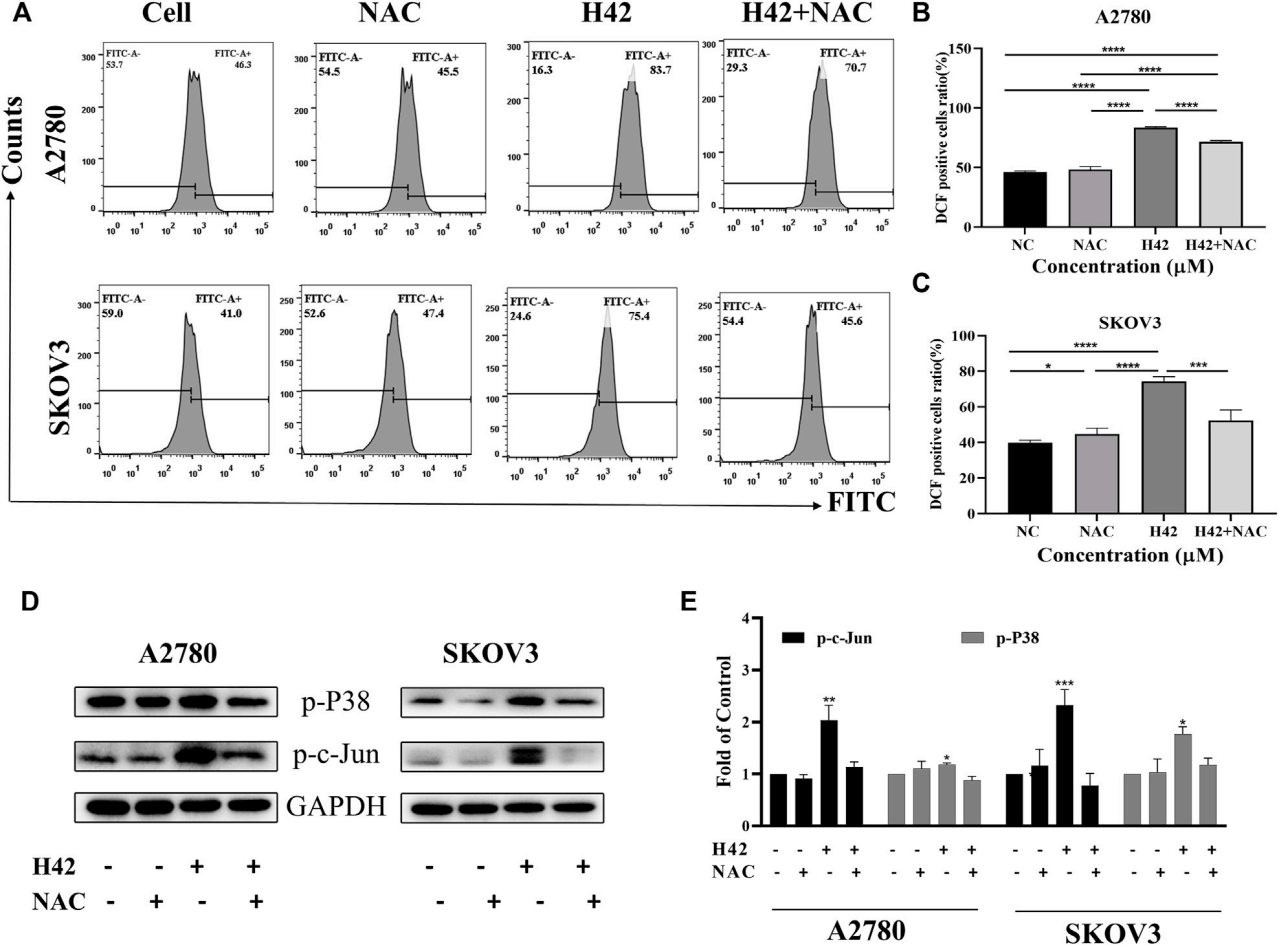
Compound H42-triggered ROS in A2780 and SKOV3 cells could be reversed by the ROS scavenger NAC. A2780 and SKOV3 cells were treated with DMSO, H42, NAC, or H42 accompanied by NAC for 48 H. **(A)** Cellular ROS was detected by the DCFH-DA probe using FCM. Quantitative analysis of ROS generation is shown in **(B,C)**. **(D)** Expression of ROS-related proteins in A2780 and SKOV3 cells was examined by Western blot and **(E)** analyzed in the density ratio. Data are all represented as mean ± SD of at least three independent experiments. *p < 0.05, **p < 0.01, ***p < 0.001, and ****p < 0.0001 were compared with the control.

Considering that mitochondrion-mediated intrinsic apoptosis was closely related with the production of intercellular ROS, we employed the DCFH-DA probe to detect intercellular ROS levels by flow cytometry after H42 treatment. It showed that with the increase of H42 concentration, intercellular ROS increased significantly (Figures 5A–C). The expression levels of stress response-related proteins were also detected, and the results showed that H42 treatment remarkably increased the expression of p-P38 and p-c-Jun (Figures 5D,E; Supplementary Figure S2B). However, the expression of p-Erk1/2 in A2780 cells increased (Figures 5D,E), while it decreased in Skov3 cells (Supplementary Figure S2B). Moreover, after the addition of ROS scavenging NAC, the cellular ROS levels (Figures 6A–C) and the ROS-related protein expression (Figures 6D,E) were all reversed, indicating that compound H42 indeed induced intercellular ROS production and increased related-protein expression.

### 3.6 Compound H42 induced DNA damage through inducing DNA double-strand breaks in ovarian cancer cells

**FIGURE 7:**
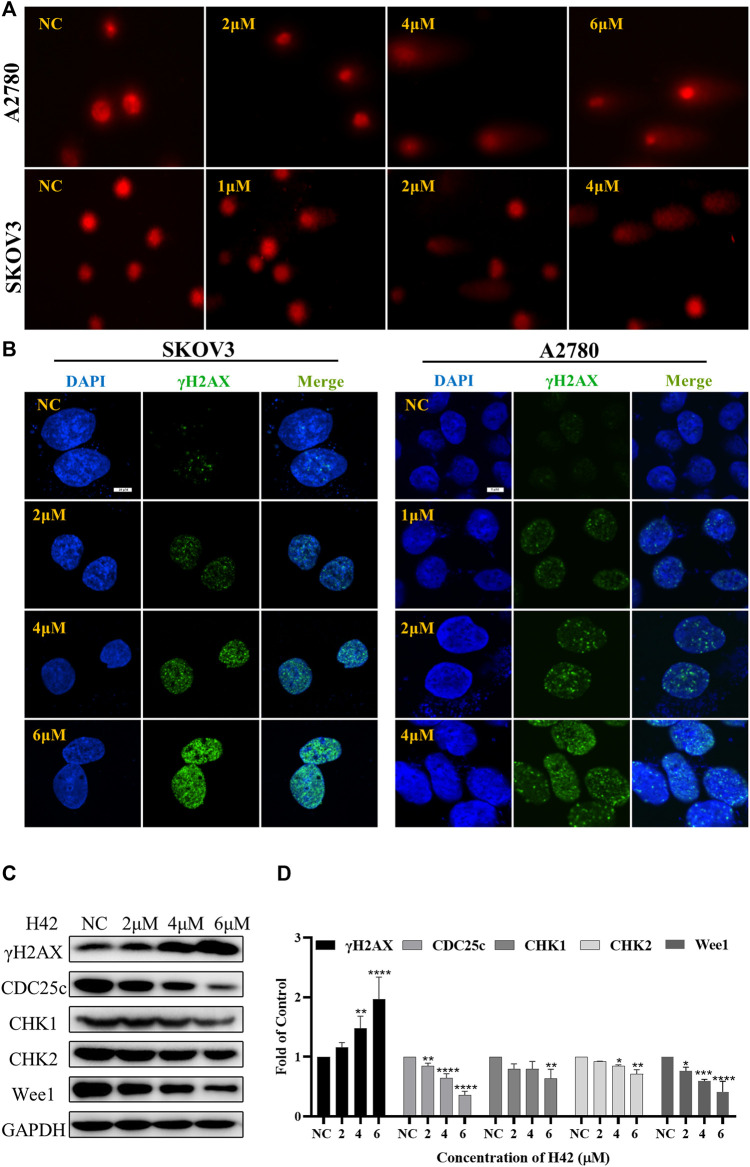
Compound H42 treatment induced DNA damage and down-regulated DNA damage response protein expression in ovarian cancer cells. A2780 and SKOV3 cells were treated with various concentrations of H42 for 48 H. **(A)** DNA damage was analyzed by alkaline comet assay. **(B)** Expression of γH2AX was detected by immunofluorescence using confocal microscopy. **(C)** Expression of DNA damage response-related proteins in A2780 cells was measured by Western blot and **(D)** analyzed in the density ratio. Data are all represented as mean ± SD of at least three independent experiments. *p < 0.05, **p < 0.01, ***p < 0.001, and ****p < 0.0001 were compared with the control.

It is previously reported that excessive ROS accumulation may induce DNA damage (Wang et al., 2012). To determine whether compound H42 exerts the same activity, alkaline comet assay was employed to detect the effect of compound H42 on DNA. The presence of a DNA tail with a greater tail area and longer tail length indicated more extensive DNA damage. A2780 and SKOV3 cells were treated with variable concentrations of compound H42 for 48 h and then subjected to comet assay. The results showed that compound H42 treatment increased DNA tail area and length, suggesting H42 treatment induced DNA strand breaks in both cell lines (Figure 7A).

To further confirm the effect of compound H42 on DNA damage, immunofluorescence (IF) was employed to monitor the phosphorylation of H2AX (γH2AX), a sensitive marker of DNA double-strand breaks (DSBs), in the nucleus of H42-treated A2780 and SKOV3 cells. Obviously, it was shown that H42 significantly increased the number of cells with γH2AX foci in the two ovarian cancer cells (Figure 7B). In addition, the results of Western blot also showed that compound H42 induced the expression of γH2AX in a concentration-dependent manner (Figures 7C,D), indicating that H42 induced DNA lesions in ovarian cancer cells. Then, we examined the expression of DNA damage response proteins CHK1, CHK2, Wee1, and p-CDC25c in A2780 cells. Compound H42 treatment caused a prominent decrease of CHK1, CHK2, Wee1, as well as p-CDC25c compared to the control groups (Figures 7C,D). The aforementioned results suggested that compound H42 treatment induced DNA damage as well as downregulation of DNA damage response proteins.

### 3.7 Compound H42 induced cell cycle arrest at the G0/G1 phase

**FIGURE 8:**
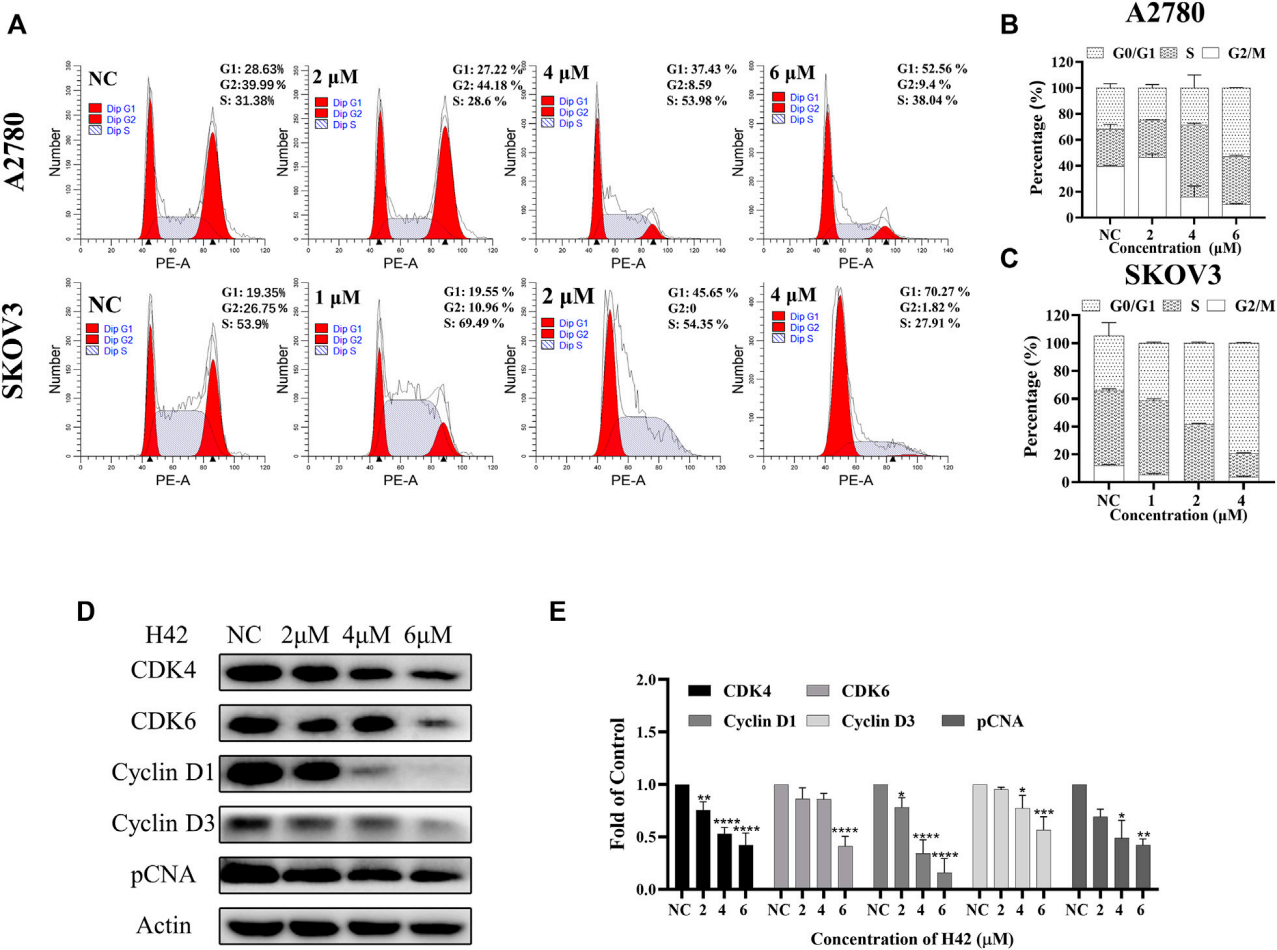
Compound H42 triggered G0/G1 cell cycle arrest in ovarian cancer cells. A2780 and SKOV3 cells were treated with various concentrations of H42 for 24 H. **(A)** Cell cycle distributions were determined by PI staining using FCM. The quantification of A2780 and SKOV3 cell cycle distributions is presented in **(B,C)**. **(D)** Expression of cell cycle-related proteins in A2780 cells was examined by Western blot and **(E)** analyzed in the density ratio. Data are all represented as mean ± SD of at least three independent experiments. *p < 0.05, **p < 0.01, ***p < 0.001, and ****p < 0.0001 were compared with the control.

To determine the underlying mechanism by which compound H42 inhibits ovarian cancer cell proliferation and apoptosis, the cell cycle distribution was analyzed in both cell lines after H42 treatment. As compared to the vehicle (ratio of the G0/G1 phase is 28.63% of A2780 cells and 19.35% of SKOV3 cells), treatment of H42 for 24 h led to an increase of the G0/G1 phase of the cell cycle to 52.56% and 70.27%, respectively, indicating that H42 could efficiently increase the percentage of cell cycle arrest at the G0/G1 phase (Figures 8A–C), while with the increase of compound H42, the number of cells in G2/M phase of the cell cycle decreased apparently (Figures 8A–C). Furthermore, the expression levels of cell cycle regulation proteins were examined. As shown in Figures 8D,E and S2 C, the expression of cell cycle promoters cyclin D1, cyclin D3, CDK4, and CDK6 was concentration-dependently reduced after H42 treatment. Meanwhile, pCNA protein expression also decreased. Taken together, these results demonstrated that compound H42 inhibited cell cycle progression *via* arresting cells at the G0/G1 phase.

### 3.8 Compound H42 induced cyclin D1 degradation in ovarian cancer cells

**FIGURE 9:**
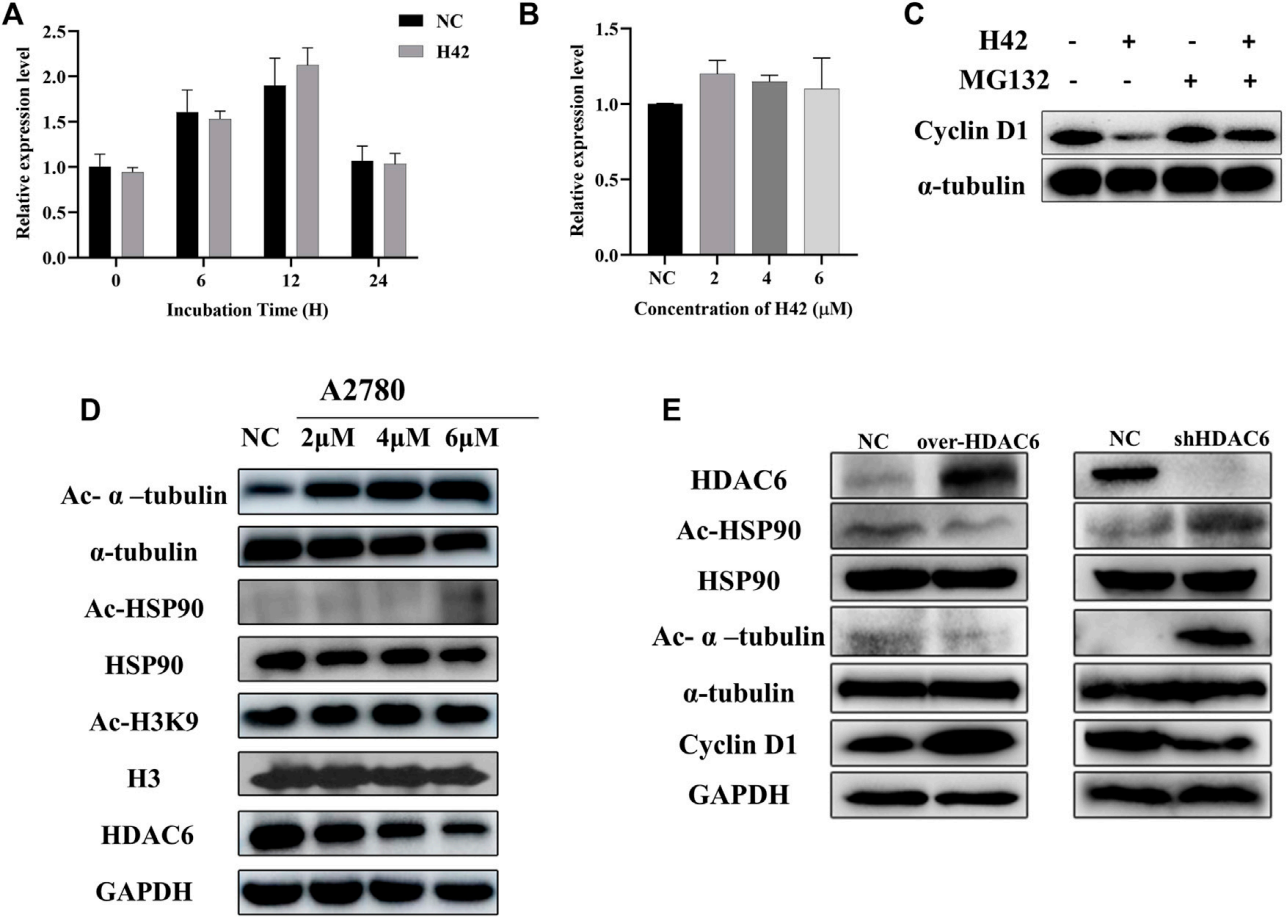
Compound H42 induced the degradation of cyclin D1 *via* HDAC6. **(A)** mRNA levels of cyclin D1 at specific points were detected by qPCR. **(B)** mRNA levels of cyclin D1 after different concentrations of H42 treatment were detected by qPCR. **(C)** H42 promoted cyclin D1 degradation. **(D)** H42 downregulated HDAC6 expression and upregulated the acetylation of α-tubulin and HSP90 in ovarian cancer cells. **(E)** Protein expression was detected in HDAC6 overexpression ovarian cancer cells and shHDAC6 A2780 cells.

It is reported that cyclin D1 played a critical role in cell cycle control (Montalto and De Amicis, 2020) and tumor development (Tashiro et al., 2007), and downregulation of cyclin D1 induced cell cycle arrest at the G1 phase (Masamha and Benbrook, 2009). Here, we noticed that compound H42 downregulated cyclin D1 expression (Figures 8D,E). Then, we further detected cyclin D1 transcription at different incubation times. A2780 cells starved for 24 h without FBS were treated with compound H42 or vehicle for up to another 24 h. The results showed that the transcription of cyclin D1 increased at 6 h and decreased at 12 h and 24 h in both H42 treatment or control groups (Figure 9A). Then, we further detected the transcription of cyclin D1 after H42 treatment at different concentrations. The results showed that when compared to the control group, there was no significant difference in the transcription of cyclin D1 among different groups (Figure 9B). The aforementioned results indicated that compound H42 treatment did not affect the transcription of cyclin D1. Next, we detected whether compound H42 affects the degradation of cyclin D1 in the presence of proteasome inhibitor MG132. The results showed that co-treatment with MG132 induced a prominent restoration of the expression of cyclin D1 (Figure 9C), suggesting that compound H42 downregulated the expression of cyclin D1 through promoting cyclin D1 degradation.

### 3.9 Compound H42 exerted inhibitory activity through HDAC6 in ovarian cancer cells

It has been demonstrated that downregulation of HDAC activity gives rise to G1 cell cycle arrest, and the benzamide group in compound H42 was similar to the zinc-binding group in HDAC6 inhibitors. We wonder whether compound H42 functions through HDAC6? To confirm the effect of H42 on HDAC6, A2780 cells were treated with compound H42 for 48 h, and the expression levels of HDAC6, acetylated α-tubulin, and HSP90 were detected by Western blot. With the increasing concentration of H42, acetylated α-tubulin and HSP90, which were mediated by HDAC6, accumulated significantly (Figure 9D; Supplementary Figure S2D). Meanwhile, the expression of HDAC6 decreased significantly (Figure 9D; Supplementary Figure S2D). However, the acetylation of histone H3, a major substrate of HDACs I, did not change, at least at K9 (Figure 9D; Supplementary Figure S2D). These findings suggested that H42 has no effect on HDACs I but decreased the expression and function of HDAC6, leading to hyper-acetylation of HSP90 and α-tubulin. It is reported that acetylation of HSP90 disturbed cyclin D1/HSP90 binding (Kim et al., 2011; Pai et al., 2015), resulting in the degradation of cyclin D1, which was consistent with our results (Figures 9A–C), suggesting that H42 may promote the degradation of cyclin D1 *via* inhibiting HDAC6-mediated deacetylation.

To further determine the relationships between compound H42-induced cyclin D1 degradation and the HDAC6 signaling pathway, A2780 cells were transfected with the HDAC6 overexpression plasmid and the related-protein expression was analyzed. The results showed that overexpression of HDAC6 induced deacetylation of HSP90 and upregulation of cyclin D1 (Figure 9E), which was contrary to that of H42 treatment. However, in HDAC6 knockdown A2780 cells, HSP90 was acetylated and α-tubulin up-regulated and cyclin D1 expression decreased, demonstrating that down-regulation of HDAC6 produced a similar effect to H42. The aforementioned results suggested that H42 increased the acetylation of HSP90 *via* regulating the function of HDAC6, which further interrupted the binding of HSP90 with cyclin D1, promoting the degradation of cyclin D1, resulting in cell growth arrest at G0/G1.

### 3.10 Compound H42 inhibited tumor growth of human ovarian cancer cells in a nude xenograft mouse model

**FIGURE 10:**
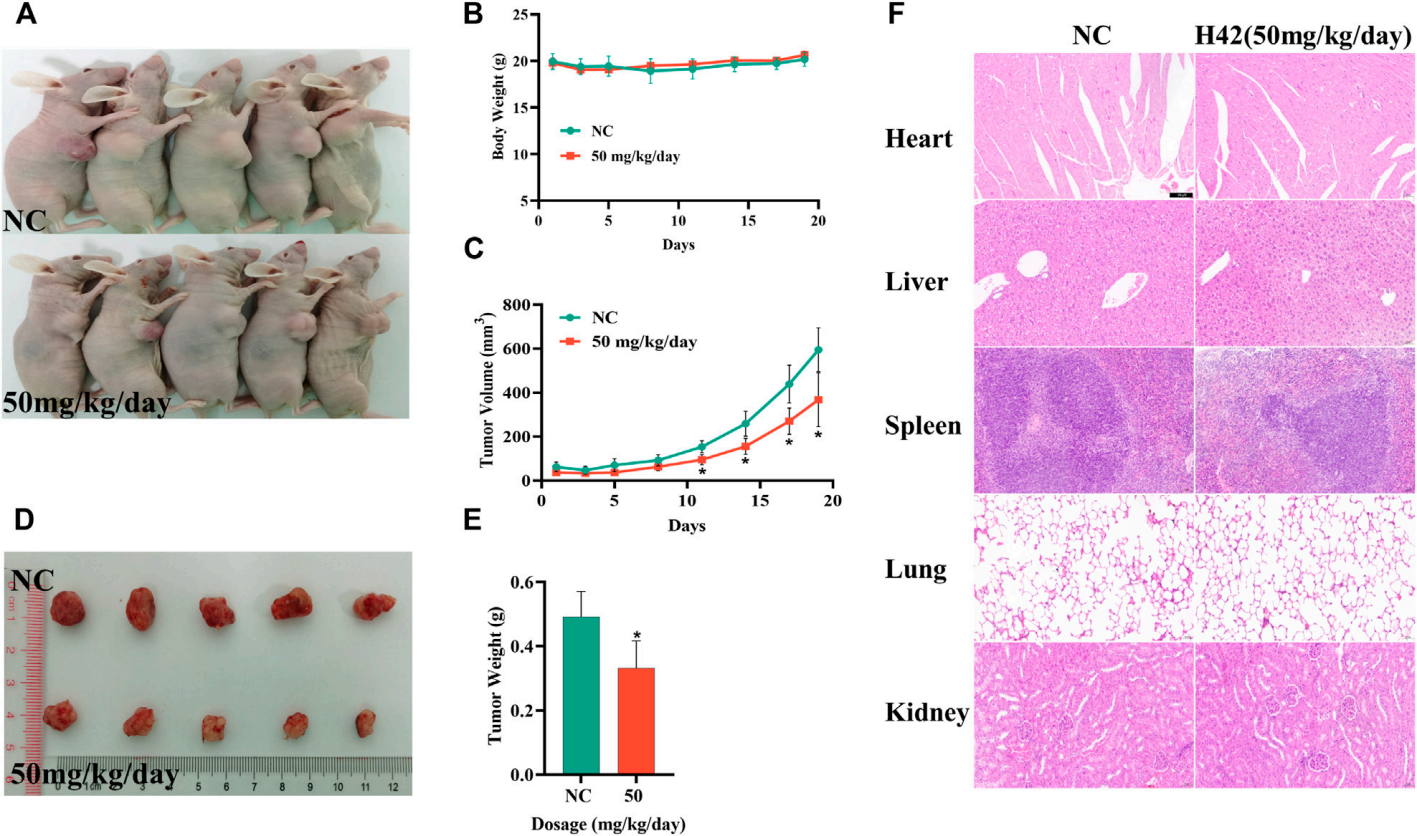
Compound H42 inhibited ovarian cancer progression *in vivo*. **(A)** Images of mice. **(B)** Monitoring of the mice body weights in each group. **(C)** Monitoring of tumor volumes in each group. **(D)** Images of tumors. **(E)** Analysis of final tumor weight. **(F)** HE staining of the heart, liver, spleen, lungs, and kidneys. The data are presented as mean ± SD. *N* = 5 per group. *p < 0.005 compared with the control group.

To further investigate the anti-tumor activity of compound H42 *in vivo*, SKOV3 cells (2 × 10^6^) were injected subcutaneously into the right axilla of female Balb/c nude mice to construct an ovarian cancer xenograft tumor model. When the tumor volume was greater than 100 mm^3^, the mice were randomly divided into two groups: one group of mice received H42 at 50 mg/kg once a day, while the control group only received PBS. The mice were treated for 19 days, and the mouse state, body weight, and tumor volume were monitored. During the treatment, there was no significant change in the body weight, and the mice were in good mental state (Figures 10A,B), suggesting that compound H42 has low toxicity to the mice. However, for the first few days, there was no significant difference in the growth rate between the two groups. Until the 11th day, the growth rate in the H42 treatment group became lower than that in the control group. The average tumor volume in the H42 treatment group was significantly smaller than that in the control group until the last day of observation (Figure 10C). Then, all the mice were euthanized, and the tumors were harvested and weighed. It was shown that the average tumor weight in the H42 treatment group was significantly lower than that in the control group (Figures 10D,E). Additionally, the results of HE staining showed no obvious damage in the heart, liver, spleen, lungs, and kidneys of the two groups (Figure 10F). The aforementioned results indicated that compound H42 has inhibitory effects on ovarian tumor growth *in vivo* without obvious toxicity.

## 4 Discussion

HDAC6 is a widely studied member of the HDACs, which is known to anchor in the cytoplasm and catalyze the deacetylation of α-tubulin, HSP90, and so on (Hubbert et al., 2002; Hai and Christianson, 2016; Ustinova et al., 2020). We found that the expression of HDAC6 decreased when cells were exposed to a high concentration of compound H42. Considering that H42 increased the acetylation of α-tubulin by dozens of times, while the expression of HDAC6 decreased at high concentrations, H42 may not only reduce its expression but also inhibit the activity of HDAC6. This phenomenon has also been reported in other HDACIs (Ahn et al., 2012; Tae et al., 2018; Mustafa et al., 2021). The hyper-acetylation of HSP90 may also promote the degradation of HDAC6, but further study will be needed.

ROS also represented an important signaling molecule participating in multiple cellular processes. The stimuli of oxidative stress and free radicals can facilitate the formation of DSBs and mitochondrion-mediated intrinsic pathway of apoptosis (Konstantinopoulos et al., 2014). So, we detected intracellular ROS in ovarian cancer cells exposed to H42 and found that H42 increased cellular ROS production in a dose-dependent manner, as previously reported in other HDACIs (Conti et al., 2010). Additionally, p-P38 and p-c-Jun were all elevated in H42 treatment groups. However, co-treatment of NAC and H42 decreased the intracellular ROS production and the phosphorylation levels of p-P38 and p-c-Jun significantly, suggesting H42 treatment increased ROS production through the MAPK signaling pathway, thereby resulting in the DBSs and apoptosis. Considering that p-Erk1/2 can mediate cell proliferation, its upregulation in H42-treated A2780 cells may lead to H42 resistance. In addition, a high level of cellular ROS leads to DNA damage, which was further exacerbated by the downregulation of DNA damage response proteins. The observation that compound H42 treatment induced a remarkable increase in γH2AX, which started before significant cell death was observed, indicates that DNA damage contributed to the treatment.

It is reported that HDACI treatment often induced the upregulation of P21 (Ueda et al., 2007; Wang et al., 2012), which could inhibit cell cycle progression. However, in this work, we found that the expression of P21 was downregulated after compound H42 treatment (data not shown). Some studies have clarified that HDACI-mediated P21 upregulation is due to the accumulating acetylation of H3 and H4 associated with the P21 gene promoter (Kim et al., 2004). Moreover, the abrogation of P21 upregulation was also observed in cancer cells treated with HDACIs accompanied with PI3K inhibitors (Rahmani et al., 2014), suggesting that H42 may function elsewhere. However, the precise mechanism needs further exploration. In addition, some researchers also reported that P21 seemed to play an anti-apoptotic role in cells treated with HDACIs (Burgess et al., 2001; Rahmani et al., 2003).

In this work, the A2780 cell line derived from ovarian endometrioid adenocarcinoma with wild-type TP53 and the SKOV3 cell was a TP53-mutant cell line derived from the ascites of serous ovarian cystadenoma. Compound H42 inhibited cell proliferation in the 2 cell lines regardless of the status of TP53, suggesting that H42 will be effective against a broad spectrum of tumor types, including tumors without p53 function.

## 5 Conclusion

Compound H42 inhibited ovarian cancer cell proliferation *via* HDAC6-mediated cyclin D1 degradation and suppressed ovarian cancer progression in nude xenograft mice, indicating compound H42 has a preclinical value in ovarian cancer therapeutics, warranting further investigation.

## Data Availability

The original contributions presented in the study are included in the article/Supplementary Material; further inquiries can be directed to the corresponding authors.
