# Supplementary material for: Identification of a novel pyridine derivative with inhibitory activity against ovarian cancer progression in vivo and in vitro
Source: Front Pharmacol. 2022 Nov 18;13:1064485. doi: 10.3389/fphar.2022.1064485 (PMC9715740; doi:10.3389/fphar.2022.1064485)
Supplement: Supplementary file 1 [file Presentation1.pdf]

Supplemental figure 1:

NMR spectra of compound H42.

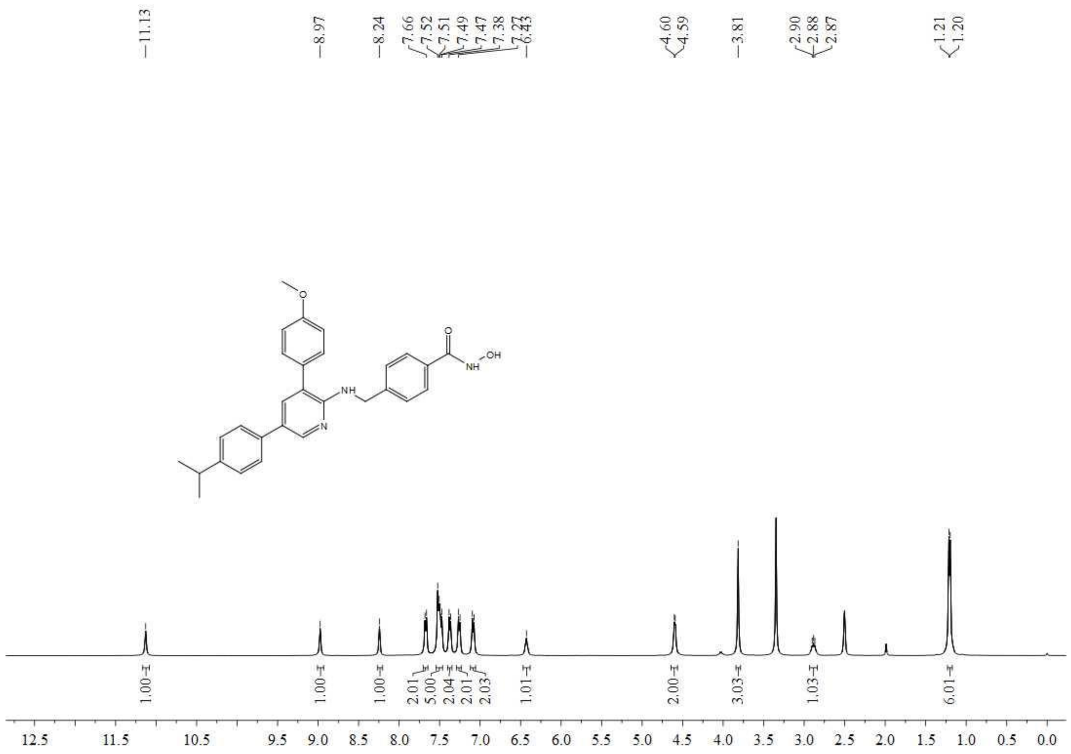

^{1}H NMR diagram of compound H42

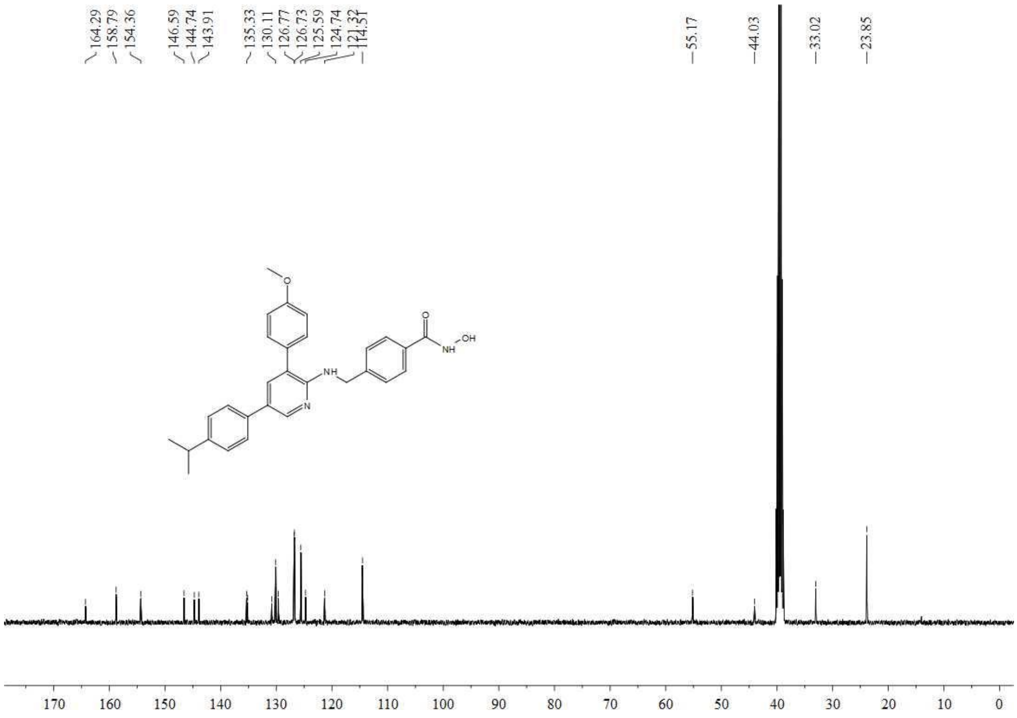

^{13}C NMR diagram of compound H42

White solid, yield 70%, MP:237.2-238.8°C, ^{1}H NMR (400 MHz, DMSO-d_6) δ 11.13 (s, 1H, OH), 8.97 (s, 1H, NH), 8.24 (s, 1H, Ar-H), 7.67 (d, J = 7.7 Hz, 2H, Ar-H), 7.50 (dd, J = 12.7, 7.9 Hz, 5H, Ar-H), 7.37 (d, J = 7.6 Hz, 2H, Ar-H), 7.26 (d, J = 7.6 Hz, 2H, Ar-H), 7.09 (d, J = 8.0 Hz, 2H, Ar-H), 6.43 (s, 1H, NH), 4.60 (d, J = 4.5 Hz, 2H, CH_2), 3.81 (s, 3H, CH_3), 2.93 – 2.84 (m, 1H, CH), 1.21 (d, J = 6.6 Hz, 6H, CH_3). ^{13}C NMR (101 MHz, DMSO-d_6) δ 164.29, 158.79, 154.36, 146.59, 144.74, 143.91, 135.33, 135.18, 130.79, 130.11, 129.62, 126.77, 126.73, 125.59, 124.74, 121.32, 114.51, 55.17, 44.03, 33.02, 23.85. HR-MS (ESI), calcd. $C_{29}H_{29}N_3O_3$, $[M+H]^+$ m/z: 468.2287, found: 468.2290.
