# Supplementary figures and images for: Identification of a novel pyridine derivative with inhibitory activity against ovarian cancer progression in vivo and in vitro
Source: Front Pharmacol. 2022 Nov 18;13:1064485. doi: 10.3389/fphar.2022.1064485 (PMC9715740; doi:10.3389/fphar.2022.1064485)

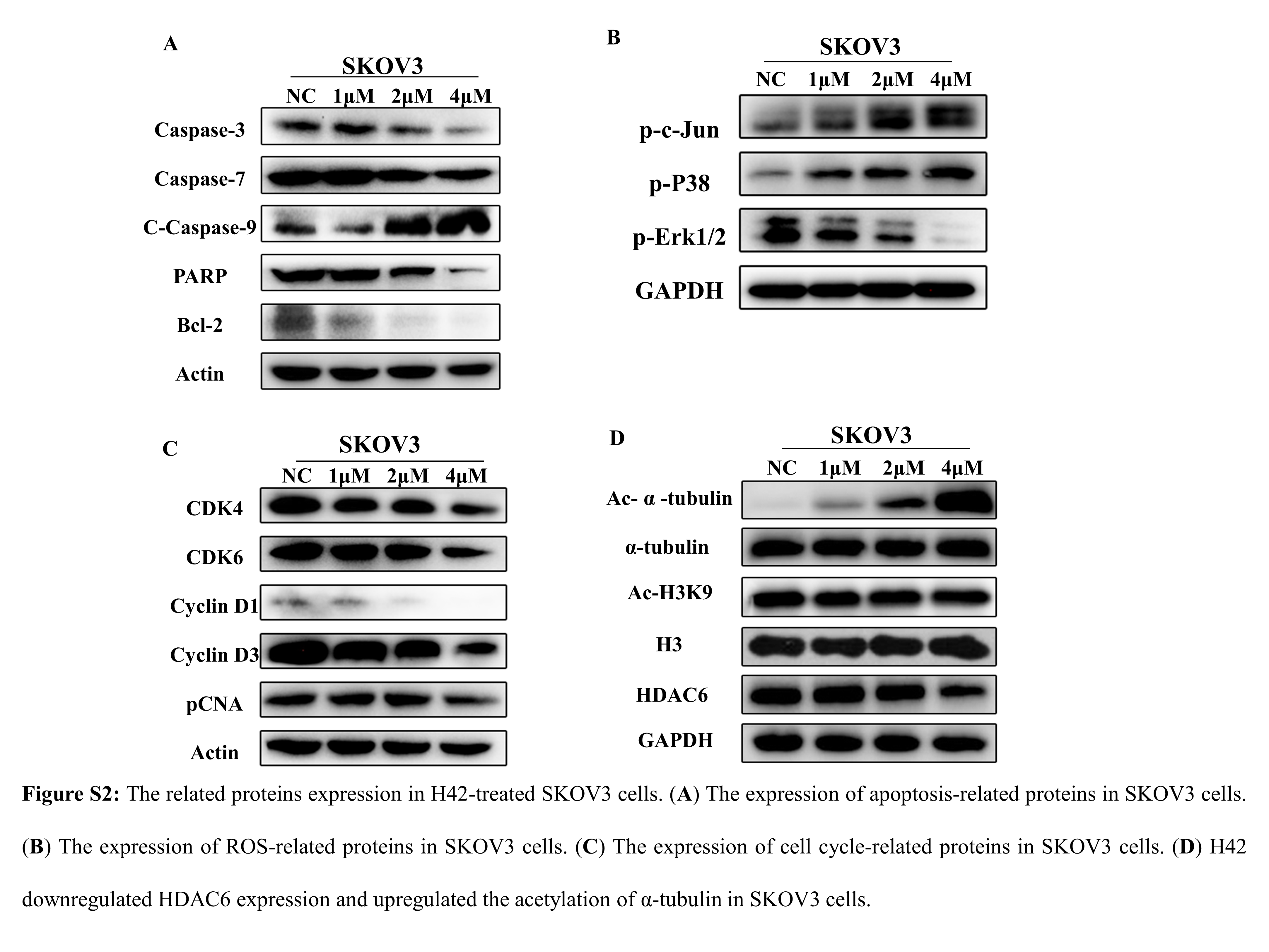

Supplement: Supplementary file 2 [file Image1.TIF]
